# What to Do with the Appendix

**Published:** 1902-03-01

**Authors:** 


### WHAT TO DO WITH THE APPENDIX.

The interest of the last meeting of the Clinical Society centred principally on the question whether or not the appendix should be removed when operating upon cases of appendicitis with abscess formation in the acute stage. Mr. Barling strongly advised that unless the appendix could be removed with ease it should be left alone. In advising this course he insisted upon the safety with which pus could be evacuated if the removal of the appendix were not insisted upon, and the rarity with which recurrence took place under such circumstances. He said that the abscess should be attacked as soon as the diagnosis of suppuration was made, and that in making this diagnosis the presence of leucocytosis was of much significance. The statistics he gave strongly bore out the propriety of the treatment which he had employed. Of 140 cases of appendicitis operated on in the acute stage, 74 had been cases of localised suppuration in the right iliac fossa or immediately adjacent to it, and in 46 of the 74 cases the abscess, though well shut off by adhesions, had not come forward so as to be adherent to the abdominal parietes, and thus the operation involved opening the peritoneal cavity. In these 74 cases there were 2 deaths. The appendix was only removed 25 times, and in 49 cases it was not removed. Mr. F. C. Wallis read a paper on the treatment of intraperitoneal abscess of the appendix in which he stated that his practice was to remove the appendix. The point of his paper however rather lay in his insistance on the fragile condition of the abscess in those cases among young people in which, although shut off by adhesions from the general peritoneal cavity, the abscess was in no way adherent to the abdominal parietes. In these cases it was necessary to open the general peritoneal cavity before the abscess could be dealt with, and great care was necessary to prevent the pus under tension in the abscess sac from escaping into the general cavity of the peritoneum. Packing with Gamgee sponges and gradual evacuation was recommended. Mr. Arthur Barker was in favour of removing the appendix at the time of the primary operation if it could be done easily, and if not then by a secondary operation in the quiescent interval. Mr. James Berry thought it was inadvisable to remove the appendix in all cases of suppurative appendicitis. In fact, even in regard to incising the abscess he seemed to advocate a waiting policy; waiting, that is, until adhesions had taken place and making a small incision. Mr. W. G. Spencer considered that it was advisable to remove the appendix at the time of the first operation. Take it altogether the general opinion seemed to be that the appendix was better removed if—always if—this could be done easily, but that it was better to let it alone than to hunt for it, which was much what Mr. Barling had said, although there was some difference of opinion as to how far the hunt should be carried, and also as to the suggested harmlessness of an appendix when left unremoved.

